# Geographical and Racial Disparities in Head and Neck Cancer Diagnosis in South-Eastern United States: Using Real-World Electronic Medical Records Data

**DOI:** 10.1089/heq.2019.0092

**Published:** 2020-03-24

**Authors:** Amrita Mukherjee, Adeniyi J. Idigo, Yuanfan Ye, Howard W. Wiener, Ravi Paluri, Lisle M. Nabell, Sadeep Shrestha

**Affiliations:** ^1^ Department of Epidemiology, School of Public Health, University of Alabama at Birmingham, Birmingham, Alabama.; ^2^ O'Neal Comprehensive Cancer Center, University of Alabama at Birmingham, Birmingham, Alabama.; ^3^ School of Medicine, University of Alabama at Birmingham, Birmingham, Alabama.

**Keywords:** rural–urban, racial disparities, age at diagnosis, head and neck cancer, epidemiology

## Abstract

**Background:** Rurality, race, and age at diagnosis are important predictors in head and neck cancer (HNC) prognosis. However, literature on the associations of rurality and race with age at HNC diagnosis is limited. Data on geographical, racial, and gender disparities in young HNC patients (diagnosed ≤45 years) are also scarce.

**Materials and Methods:** This retrospective study assesses rural–urban, racial, and gender disparities in age at HNC diagnosis, using electronic medical records (Cerner) data of 4258 HNC patients (1538 residing in rural counties and 2720 in urban counties) from National Cancer Institute-designated cancer center in Alabama. Rurality was defined based on 2010 U.S. Census Bureau's rural–urban classification. Logistic regression was used to assess the association of young HNC diagnosis with demographical, behavioral, and clinical variables. ArcGIS 10.2 was used to map geospatial distribution of age and population-adjusted HNC case across rural and urban counties.

**Results:** Patients from rural counties were less likely to be diagnosed at younger age (≤45 years) compared with urban counties (odds ratio [OR] [95% confidence interval (CI)]: 0.74 [0.58–0.93]). Most patients present at stage III/IV (64.9% in rural and 60.2% in urban). Compared with white patients, black patients were 70% more likely to get diagnosed at a young age (95% CI: 1.23–2.35). Young patients were more likely to be females and blacks compared with older patients (*p*<0.0001). Among oral cavity cancer patients, rural patients were 51% less likely to get diagnosed at young age compared with urban patients (95% CI: 0.27–0.89).

**Conclusions:** Head and neck cancer screening is not routinely conducted so most show up at later stage of cancer. There is also evidence of disparities in age at HNC diagnosis based on rurality, race, and gender; targeted screening can help in reducing these disparities.

## Introduction

Head and neck cancer (HNC) is one of the common malignancies affecting people worldwide. Approximately 650,000 HNC cases and 380,000 HNC-related deaths are reported annually worldwide.^1,2^ In the United States, 3% of all malignancies and 13,000 annual deaths can be attributed to HNC.^3^ Males are more likely to be affected by HNC compared with females, with a male-to-female HNC patient ratio of 3:1.^4^ However, over the past couple of decades, there is evidence of a decline in the incidence of tobacco- and alcohol-associated HNCs, such as cancers of the oral cavity, hypopharynx, and larynx in men; and an increase in the incidence of human papillomavirus (HPV)-associated oropharyngeal cancer (OPC) and Epstein Barr virus (EBR)-associated nasopharyngeal cancer.^5–7^ HNC malignancies, previously thought of as a disease affecting mostly white males in their 6th/7th decades of life, are now on the rise among young patients of both genders.^8,9^

With advancements in HNC treatment modalities and increase in incidence of HPV-associated OPC cases, survival among HNC patients has improved over the past two decades.^10^ Epidemiological shifts have also been reported, as there is an increase in the proportion of young, mostly male patients diagnosed with HPV-associated OPC, compared with traditionally older HNC patients.^5,11^ An increased number of oral sex partners have also attributed to the increase in HPV-associated OPC among young individuals.^11,12^ On the contrary, increased incidence of oral cavity cancer, especially tongue cancer, has been reported in young women; though the etiology is still not clear.^5,8,13,14^ To date, only limited studies have focused on young HNC patients, despite the rise of HNCs in the young. No studies have focused on the effect of rural–urban disparities on age at HNC diagnosis.^8^

There is conflicting evidence on the effect of rurality on HNC incidence and prognosis. Some studies have shown that HNC patients residing in urban settings have better overall and disease-free survival compared with patients in rural settings,^15,16^ and these findings have been attributed to factors such as differential access to health care, physician density, primary treatment choice, and socioeconomic status.^17^ Other studies have reported increased incidence, adverse outcomes in urban dwellers,^18^ or no rural–urban disparities.^19^ Age at diagnosis is an important prognostic factor in all types of cancers, and HNC is no exception. Some studies report better prognosis in young patients,^20–22^ while others report differently.^23^ Although there is no consensus on the definition of “young” patients, most studies have defined patients aged ≤45 years as “young.”^8^ The objectives of this study was to evaluate if rural–urban disparities exist overall, and in age at HNC diagnosis among patients diagnosed or presenting with HNC at the Comprehensive Cancer Center (CCC) at the University of Alabama at Birmingham (UAB), with a focus on young patients (aged ≤45 at the time of HNC diagnosis). UAB CCC, being the only National Cancer Institute (NCI) designated center in Alabama, has a large cancer patient catchment area, including Mississippi and Louisiana. Disparities in age at diagnosis based on race, gender, and anatomic subsites were assessed in HNC patients.

## Materials and Methods

### Study population

For this retrospective study, 4258 patients diagnosed or presenting with incident HNC at the UAB CCC between January 2012 and March 2018 were included. Patients were identified from the UAB electronic medical records (Cerner) databases using International Classification of Diseases (ICD) codes versions 9 and 10 (ICD 9/10). ICD9 codes included in the study were 140.–149., 160. (except 160.1), 161., and 195.0. ICD10 codes included were C00–C14, C30, C31, C32, and C76.0. Patients ≥18 years who were residents of Alabama at the time of incident primary HNC diagnosis were included in the analysis. This study was approved by the UAB Institutional Review Board, as well as by the CCC.

### Residential setting

The primary exposure of interest was rurality. Rural and urban areas were defined based on 2010 U.S. Census Bureau's urban–rural classification.^24^ Urban areas were defined as areas with ≥50,000 residents; urbanized clusters were defined as areas with ∼2500 but <50,000 people; areas that did not belong to either urban or urbanized clusters designations were defined as rural.^24^ Counties were classified based on the 2010 U.S. Census Bureau's rurality classification. Counties with <50% of the population living in rural areas were classified as “mostly urban”; 50–99.9% of population living in rural areas as “mostly rural,” and counties where 100% of the population lived in rural areas were classified as “Completely rural.”^24^ Further, “mostly rural” and “completely rural” counties were combined into one category—“Rural” and the “mostly urban” counties formed the “Urban” counties category.

### Age at incident HNC diagnosis/presentation

Age at incident HNC diagnosis/presentation at UAB in general and also at “Rural” and “Urban” settings was of primary interest. For patients who were diagnosed with primary HNC at UAB, age at diagnosis was recorded. However, for patients who were diagnosed elsewhere, but were referred to UAB for HNC treatment and management, age at first HNC presentation at UAB was recorded. Since UAB is the only NCI-designated cancer center in Alabama, it was assumed that age at cancer presentation for referred patients would not differ much from their actual age at diagnosis. Henceforth, term “age at diagnosis” was used for all patients. Age at diagnosis was assessed both as a continuous variable and as a dichotomous variable young (≤45 years) and old (>45 years), as defined by previous studies.

### Other covariate measures

Other variables of interest were race, gender, and HNC anatomic subsites. Race was self-reported and categorized as whites, blacks, and other races. Gender included male and female. HNC patients were categorized based on the following anatomic subsites: oral cavity, oropharynx, nasopharynx, larynx and hypopharynx, nasopharynx, nasal cavity and paranasal sinuses, major salivary glands and HNC of unspecified origin (cancer in multiple sites, other ill-defined parts of head and neck including unspecified parts of nasal cavity, oral cavity or floor of mouth, or carcinoma of unknown primary). Other covariates included marital status, smoking status, alcohol status, insurance status, stage at cancer diagnosis, and vital status. Clinical and pathological staging, classified based on the American Joint Committee on Cancer TNM classification, 7th edition^25^ was obtained from the UAB Cancer Registry. Patients were categorized by combining stages III and IV into one category and stages I and II into another category.

### Statistical analysis

Normality assumptions were checked for all continuous variables. Univariate statistics were reported using mean (±standard deviation), median (interquartile range [IQR]), or frequency (%) as appropriate. For bivariate statistics, chi-square and Wilcoxon statistics were reported. Logistic regression model assumptions were checked; unadjusted and adjusted logistic models were reported. Probability of being diagnosed at young age was modeled on race, gender, smoking status, alcohol status, anatomic subsites, and rurality. Variables with an unadjusted association of *p*<0.10 were included in the multivariable model. Association of rurality with age at diagnosis was also assessed separately in patients with oral cavity, OPC and larynx cancer, the most common anatomic subsites involved in HNC, specifically in head neck squamous cell carcinoma. Sensitivity analysis was performed in patients with information available on HNC staging at diagnosis. Statistical significance was set at *p*≤0.05. Odds ratio (OR), 95% confidence interval (95% CI), and two-sided *p*-values were reported. All statistical analyses were conducted in SAS 9.4 (Cary, NC). Age- and population-adjusted geospatial distribution of HNC cases by rural–urban counties in Alabama was also mapped using ArcGIS 10.2 (Redlands, CA).

## Results

**FIG. 1.**
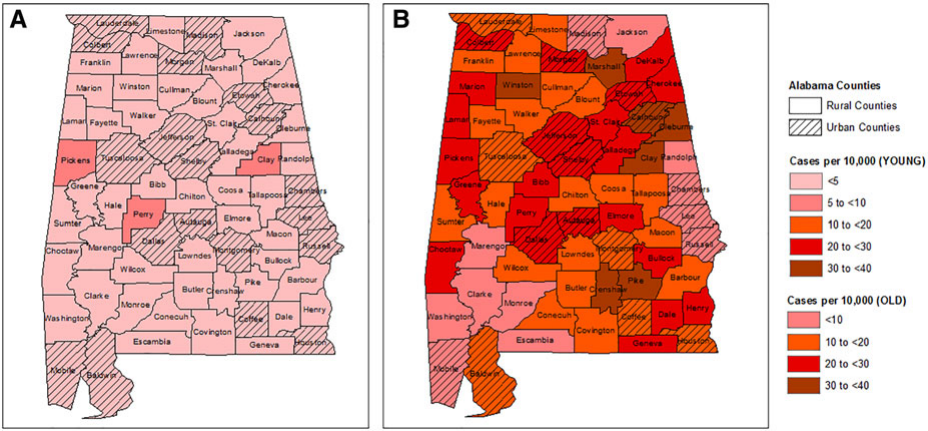
Geospatial distribution of HNC cases (per 10,000 people) in Alabama counties by age group, using age-adjusted reference population data from U.S. Census 2010; **(A)** HNC cases per 10,000 people in the young group (diagnosis age ≤45 years) and **(B)** HNC cases per 10,000 people in the old group (diagnosis age >45 years). Rural and urban counties are defined based on 2010 U.S. Census Bureau's rurality classification. HNC, head and neck cancer.

Of the 4258 HNC patients, 68.9% were males and 73.7% were white, 14.6% black and 11.7% of other races (Table 1). A total of 1538 resided in rural counties, and the rest 2720 patients came from urban counties. Median age at HNC diagnosis for the entire adult study population was 63.0 years ([IQR]: 55.0–72.0 years). Patients residing in urban counties were diagnosed slightly at a younger age (median age in years [IQR]: 62.2 [54.0–72.0]) compared with patients from rural counties (median age [IQR]: 63.2 [56.0–72.0], [*p*=0.03]). Statistically significant differences were observed in the distribution of race between the rural and urban patients (*p*<0.0001). Compared with 9.7% black patients in rural counties, 17.3% of the patients in urban counties were black. A higher proportion of patients in the rural counties were current or former smokers, compared with the proportion of patients in the urban counties (67% in rural vs. 60.9% in urban counties, [*p*=0.004]). Proportion of patients consuming alcohol (current users) at the time of HNC diagnosis was higher in urban counties compared with rural (33.3% vs. 26.2%, *p*<0.0001). Oral cavity was the most commonly affected anatomic subsite overall (24.2%), as well as in both geographical groups. Geospatial distribution of age of HNC cases indicated some hotspots of young and old patients (Fig. 1).

**Table 1.** Demographical, Behavioral, and Clinical Characteristics of Head and Neck Cancer Patients Diagnosed at the University of Alabama at Birmingham Comprehensive Cancer Center between January 2012 and March 2018 by Urban–Rural Counties in Alabama

Exposure/covariates	All patients	Rural counties	Urban counties	p^a^
	(*n*=4258)	(*n*=1538)	(*n*=2720)	
	Frequency (%)	Frequency (%)	Frequency (%)	
Age at cancer presentation/diagnosis in years				
Median (IQR)	63.0 (55.0–72.0)	63.2 (56.0–72.0)	62.2 (54.0–72.0)	0.03
Gender				
Male	2933 (68.9)	1083 (70.4)	1850 (68.0)	0.10
Female	1325 (31.1)	455 (29.6)	870 (32.0)	
Race				
White	3136 (73.7)	1236 (80.4)	1900 (69.9)	<0.0001
Black	622 (14.6)	150 (9.7)	472 (17.3)	
Other	500 (11.7)	152 (9.9)	348 (12.8)	
Marital status				
Married/with partner	2225 (52.3)	843 (54.8)	1382 (50.8)	0.07
Divorced/separated/widowed	750 (17.6)	265 (17.2)	485 (17.8)	
Single	823 (19.3)	273 (17.8)	550 (20.2)	
Unknown	460 (10.8)	157 (10.2)	303 (11.1)	
Smoking status				
Current	771 (26.9)	297 (29.4)	474 (25.6)	0.004
Former	1035 (36.2)	380 (37.6)	655 (35.3)	
Never	1058 (36.9)	333 (33.0)	725 (39.1)	
Alcohol status				
Current	1180 (30.7)	362 (26.2)	818 (33.3)	<0.0001
Former	347 (9.1)	128 (9.3)	219 (8.9)	
Never	2313 (60.2)	890 (64.5)	1423 (57.8)	
Insurance status				
Private insurance	2381 (55.9)	818 (55.0))	1563 (59.5)	<0.0001
Medicare	1051 (25.5)	419 (28.2)	632 (24.1)	
Medicaid	407 (9.9)	175 (11.8)	232 (8.8)	
Self-pay/uninsured	276 (6.7)	76 (5.1)	200 (7.6)	
Anatomic subsite				
Oral cavity	1029 (24.2)	351 (22.2)	678 (24.9)	0.10
Oropharynx	813 (19.1)	283 (18.4)	530 (19.5)	
Larynx and hypopharynx	791 (18.6)	284 (18.5)	507 (18.6)	
Nasopharynx, nasal cavity, and sinuses	285 (6.7)	96 (6.2)	189 (7.0)	
Major salivary	438 (10.3)	179 (11.6)	259 (9.5)	
HNC of unspecified origin^b^	902 (21.2)	345 (22.4)	557 (20.5)	
Cancer stage at diagnosis				
Stage I/II	440 (38.0)	151 (35.1)	289 (39.8)	0.117
Stage III/IV	717 (62.0)	279 (64.9)	438 (60.2)	

^a^ *p* value based on chi-square and Wilcoxon tests for the two groups (rural vs. urban counties); missing data for alcohol status=418, smoking status=1394, insurance status=143, cancer stage at diagnosis=3101.

^b^ Cancer in multiple sites, other ill-defined parts of head and neck, including unspecified parts of nasal cavity, oral cavity or floor of mouth, or CUP.

CUP, carcinoma of unknown primary; HNC, head and neck cancer; IQR, interquartile range.

Among young HNC patients, 45.3% were females compared with 29.7% female patients (*p*<0.0001) in the old group (Table 2). Proportion of black patients in the young group was 22.2% compared with 13.8% in the old group (*p*<0.0001). In the young group, 52.3% of the participants were never smokers compared with 35.4% in the old group (*p*<0.0001). Proportion of oral cavity cancer was higher among young patients compared with old patients (29.9% vs. 23.6%). Similar trends were observed in patients with nasopharynx, nasal cavity, and paranasal sinuses cancer (13.0% in young vs. 6.1% in old patients). Compared with 36.8% old patients residing in rural counties, 29.3% of the young patients came from rural counties (*p*=0.004) (Table 2).

**Table 2.** Demographical, Behavioral, and Clinical Characteristics of Head and Neck Cancer Patients Diagnosed at the University of Alabama at Birmingham Comprehensive Cancer Center between January 2012 and March 2018 by Age at Diagnosis

Exposure/covariates	All patients	Young group	Old group	p^a^
	(*n*=4258)	(*n*=391)	(*n*=3867)	
	Frequency (%)	Frequency (%)	Frequency (%)	
Age at cancer presentation/diagnosis in years				
Median (IQR)	63.0 (55.0–72.0)	37.0 (30.0–42.0)	64.0 (57.0–73.0)	<0.0001
Gender				
Male	2933 (68.9)	214 (54.7)	2719 (70.3)	<0.0001
Female	1325 (31.1)	177 (45.3)	1146 (29.7)	
Race				
White	3136 (73.7)	263 (67.3)	2873 (74.3)	<0.0001
Black	622 (14.6)	87 (22.2)	535 (13.8)	
Other	500 (11.7)	41 (10.5)	459 (11.9)	
Marital status				
Married/with partner	2225 (52.3)	164 (41.9)	2061 (53.3)	<0.0001
Divorced/separated/widowed	750 (17.6)	30 (7.7)	720 (17.6)	
Single	823 (19.3)	172 (44.0)	823 (19.3)	
Unknown	460 (10.8)	24 (6.4)	435 (11.3)	
Smoking status				
Current	771 (26.9)	65 (25.2)	706 (27.1)	<0.0001
Former	1035 (36.2)	58 (22.5)	977 (37.5)	
Never	1058 (36.9)	135 (52.3)	923 (35.4)	
Alcohol status				
Current	1180 (30.7)	121 (33.7)	1059 (30.4)	0.37
Former	347 (9.1)	28 (7.8)	319 (9.2)	
Never	2313 (60.2)	210 (58.5)	2103 (60.4)	
Insurance status				
Private insurance	2381 (55.9)	265 (70.7)	2116 (56.6)	<0.0001
Medicare	1051 (25.5)	26 (6.9)	1025 (27.4)	
Medicaid	407 (9.9)	47 (12.5)	360 (9.6)	
Self-pay/uninsured	276 (6.7)	37 (9.9)	239 (6.4)	
Anatomic subsite				
Oral cavity	1029 (24.1)	117 (29.9)	912 (23.6)	<0.0001
Oropharynx	813 (19.1)	60 (15.4)	753 (19.5)	
Larynx and hypopharynx	791 (18.6)	48 (12.3)	743 (19.2)	
Nasopharynx, nasal cavity, and sinuses	285 (6.7)	51 (13.0)	234 (6.1)	
Major salivary	438 (10.3)	53 (13.5)	385 (10.0)	
HNC of unspecified origin^b^	902 (21.2)	62 (15.9)	840 (21.7)	
Cancer stage at diagnosis				
Stage I/II	440 (38.0)	19 (27.1)	421 (38.7)	0.057
Stage III/IV	717 (62.0)	51 (72.9)	666 (61.3)	
Rurality				
Rural counties	1538 (36.1)	115 (29.3)	1423 (36.8)	0.004
Urban counties	2720 (63.9)	276 (70.4)	2444 (63.2)	

^a^ *p*-value based on chi-square and Wilcoxon tests for the two groups (young vs. old), young group: age at diagnosis ≤45 years, old group: age at diagnosis >45 years.

^b^ Cancer in multiple sites, other ill-defined parts of head and neck, including unspecified parts of nasal cavity, oral cavity or floor of mouth, or CUP. Missing data for alcohol status=418, smoking status=1394, insurance status=143, cancer stage at diagnosis=3101.

In the unadjusted model, females were ∼2 times (OR [95% CI]: 1.96 [1.59–2.42]) as likely to be diagnosed at a young age compared with males (Table 3). Black patients had 78% higher odds (OR [95% CI]: 1.78 [1.37–2.30]) of being diagnosed at young age compared with white patients. Patients from rural counties had 28% reduced odds of being diagnosed at a young age (OR [95% CI]: 0.72 [0.57–0.90]) compared with patients from urban counties. Compared with never smokers, current and former smokers had significantly reduced odds of getting diagnosed at a young age in the unadjusted model (OR [95% CI]: 0.63 [0.46–0.86] and 0.41 [0.30–0.56], respectively). Patients with oropharynx, larynx and hypopharynx, and unspecified HNC site cancer had reduced odds of being diagnosed at young age compared with oral cavity cancer patients. However, patients with nasopharynx cancer had 1.70 times the odds of getting diagnosed at a young age compared with oral cavity cancer patients (OR [95% CI]: 1.70 [1.19–2.43]). In the fully adjusted model, similar associations persisted for gender and race, after adjusting for smoking status, anatomic subsites, and rurality. Patients from rural counties had 31% reduced odds of getting diagnosed at young age, compared with patients from urban counties (OR [95% CI]: 0.69 [0.51–0.93]). Compared with never smokers, former smokers had 50% reduced odds of getting diagnosed at a young age (OR [95% CI]: 0.50 [0.36–0.69]). Patients with nasopharynx cancer were more than twice as likely to get diagnosed at a young age compared with oral cavity cancer patients (OR [95% CI]: 2.18 [1.40–3.41]).

**Table 3.** Unadjusted and Fully Adjusted Associations between Age at Head and Neck Cancer Diagnosis (Odds of Getting Diagnosed at Age ≤45 Years) and Rurality, Covariates of Interest

Exposure/covariates	Unadjusted	Adjusted
	OR (95% CI)	OR (95% CI)
Gender		
Male	Reference	Reference
Female	1.96 (1.59–2.42)^a^	2.05 (1.57–2.69)^a^
Race		
White	Reference	Reference
Black	1.78 (1.37–2.30)^a^	1.70 (1.23–2.35)^a^
Other	0.98 (0.69–1.38)	1.00 (0.63–1.59)
Smoking status		
Never	Reference	Reference
Current	0.63 (0.46–0.86)^a^	0.78 (0.56–1.08)
Former	0.41 (0.30–0.56)^a^	0.50 (0.36–0.69)^a^
Alcohol status		
Never	Reference	
Current	1.14 (0.90–1.45)	
Former	0.88 (0.58–1.32)	
Anatomic subsite		
Oral cavity	Reference	Reference
Oropharynx	0.62 (0.45–0.86)^a^	0.70 (0.47–1.06)
Larynx and hypopharynx	0.50 (0.36–0.71)^a^	0.72 (0.47–1.12)
Nasopharynx, nasal cavity, and sinuses	1.70 (1.19–2.43)^a^	2.18 (1.40–3.41)^a^
Major salivary	1.07 (0.76–1.52)	1.11 (0.71–1.74)
Other HNC of unspecified origin	0.58 (0.42–0.79)^a^	0.69 (0.46–1.03)
Rurality		
Urban counties	Reference	Reference
Rural counties	0.72 (0.57–0.90)^a^	0.69 (0.51–0.93)^a^

^a^ *p*<0.05.

CI, confidence interval; OR, odds ratio.

In both unadjusted and adjusted models (Table 4), black patients with oral cavity cancer were nearly twice as likely to get diagnosed at young age as white oral cavity cancer patients (OR [95% CI]: 2.14 [1.29–3.54] and 1.93 [1.05–3.54]), respectively. Among oral cavity cancer patients, former smokers were less likely to get diagnosed at a young age, compared with never smokers (*p*=0.04); however, it did not show any independent effect when adjusted for race and rurality. After adjusting for race and smoking status, oral cavity patients from rural counties were 51% less likely to get diagnosed at young age compared with oral cavity cancer patients from urban counties (OR [95% CI]: 0.49 [0.27–0.89]). No statistically significant differences were observed in age at diagnosis based on rurality among OPC (*p*=0.59) and larynx cancer patients (*p*=0.24) (data not shown). Information on HNC stage at diagnosis was available for 1157 patients, of which 62.0% were diagnosed at stages III/IV. A higher proportion of young patients were diagnosed at stages III/IV compared with older patients (72.9% vs. 61.3%, *p*=0.057). No statistically significant associations were observed between rurality (*p*=0.117) and race (*p*=0.115) with HNC stage at diagnosis.

**Table 4.** Unadjusted and Fully Adjusted Associations between Age at Oral Cavity Cancer Diagnosis (Odds of Getting Diagnosed at Age ≤45 Years) and Rurality, Covariates of Interest

Exposure/covariates	Unadjusted	Adjusted
	OR (95% CI)	OR (95% CI)
Gender		
Male	Reference	
Female	1.35 (0.92–1.98)	
Race		
White	Reference	Reference
Black	2.14 (1.29–3.54)^a^	1.93 (1.05–3.54)^a^
Other	1.32 (0.74–2.35)	1.25 (0.59–2.68)
Smoking status		
Never	Reference	Reference
Current	0.58 (0.32–1.04)	0.62 (0.34–1.11)
Former	0.55 (0.31–0.97)^a^	0.57 (0.32–1.02)
Alcohol status		
Never	Reference	
Current	0.94 (0.61–1.46)	
Former	0.88 (0.40–1.91)	
Rurality		
Urban counties	Reference	Reference
Rural counties	0.49 (0.31–0.78)^a^	0.49 (0.27–0.89)^a^

^a^ *p*<0.05.

## Discussion

To our knowledge, this is the first study assessing rural–urban disparities in age at diagnosis of HNC, with a focus on young adult patients. HNC patients from rural counties in Alabama were less likely to be diagnosed at a young age compared with patients from urban counties. Proportion of black HNC patients was significantly higher in urban counties and black patients were more likely to get diagnosed at young age compared with white HNC patients. Age at diagnosis is an important prognostic factor in HNC patients; however, there is no consensus in the literature on how age at diagnosis affects prognosis in HNC patients.^22,23,26^ On the contrary, differences in age at diagnosis raise questions about disparities in access to care, rural–urban distribution, health awareness, cancer screening, HNC risk factors, identifying populations at risk, and changing epidemiological trends; many of these disparities are not adequately addressed in the current literature.

Data on rural–urban disparities in HNC diagnosis, treatment, and prognosis are also limited and conflicting. Some studies have reported advanced stage at presentation and poorer outcomes among rural oral cavity cancer patients.^16,27^ On the contrary, Pagedar et al. observed advanced HNC disease presentation in urban patients compared with rural patients.^28^ Differences in primary treatment choice based on rurality among HNC patients have also been reported.^17^ However, to our knowledge, we report for the first time that rural patients are less likely to get diagnosed with HNC at young age compared with urban patients. Some of our findings are in line with studies that focused on rural–urban disparities in other types of cancers and reported delayed diagnosis, higher odds of unstaged cancer diagnosis, lower odds of screening, and poorer outcomes in rural patients compared with urban patients.^29–31^

Racial and gender disparities were observed in age at diagnosis. Compared with white HNC patients, black patients were more likely to get diagnosed at a young age. However, based on this information alone, it would be unwise to conclude if young black patients in Alabama are diagnosed with HNC at a young age due to better access to care compared with whites, or if severity of HNC stage at diagnosis has a role to play in early diagnosis in black patients. Even though previous studies have reported more advanced disease presentation in black patients compared with white patients,^32–34^ we did not observe any statistically significant differences in stage at HNC diagnosis based on race. This could partly be because we had HNC stage at diagnosis data available for only a subset of the patients in our study population. Further information on other socioeconomic factors, health care accessibility, and behavioral factors need to be considered while explaining these discrepancies. On the contrary, female HNC patients in our study were more likely to get diagnosed at age ≤45 years, compared with male patients—a finding consistent with previous studies that reported increased incidence of HNC cases among young females in western countries.^5,8,35^

Rural–urban differences were also observed in distribution of race, smoking, alcohol status, and insurance status in our study population. Urban counties reported a higher proportion of black patients compared with rural counties. This could be explained partly by racial distribution differences between rural and urban counties in Alabama, and partly by differences in behavioral risk factors and sexual practices among young patients.^5,36^ However, it is noteworthy to mention that the racial distribution of our study population was different from the racial distribution overall in the state of Alabama, as well as from the racial distribution reported among oral cavity and pharynx cancer cases in the Alabama Statewide Cancer Registry. Blacks constitute ∼26% of the population in Alabama,^37^ and only 14.6% of our study participants were black. On the contrary, compared with 73.7% whites in our study population, 82.7% of the oral cavity and pharynx cancer cases in the Alabama registry were whites.^38^ Even though the proportion of black HNC patients in our study population was not very different from the proportion reported in the SEER data,^39^ it was slightly higher than the proportion reported in the SEER data.^39^ Differences in racial distribution proportion between our study population, Alabama cancer registry, and SEER data could partly be explained by differences in calendar years and anatomic subsites. However, reasons for these racial disparities still remain unanswered. Proportion of current and former smokers was higher in rural counties. Former smokers were less likely to get diagnosed with HNC at a young age compared with never smokers; similar trends were observed with oral cavity cancer patients. Given that malignant transformation in HNC is associated with duration of tobacco exposure,^8^ we can assume that short exposure in young patients could explain the differences in age at diagnosis based on smoking status in our study population.

Another novel finding from our study is the proportion of nasopharynx and nasal cavity cancer cases and their age at diagnosis. Even though nasopharynx and nasal cavity cancer is relatively uncommon in the United States,^40^ 6.7% of the HNC patients were diagnosed with nasopharynx cancer in our study population. Nasopharynx and nasal cavity cancer patients were more likely to be diagnosed at a young age compared with other HNC anatomic subsites. This could be explained partly by a higher proportion of black patients in the young group compared with the old group in our study population, given that young blacks have the highest incidence of nasopharynx and nasal cavity cancer compared with young patients of all races.^40,41^ However, we did not have information on EBR and other environmental factors, and we did not include patients aged ≤18 years at diagnosis; so the findings need to be interpreted accordingly.

In summary, our findings highlight the uneven burden of HNC by gender, race, and rurality in Alabama. One of the major strengths of our study was using real-time EMR data from Alabama's largest university hospital, UAB. Even though our study population was not a nationally representative sample and included Alabama residents only, we had a diverse patient population because UAB is the only NCI-designated cancer center in the state. Large sample size provided us with sufficient statistical power in our analyses. While information on HPV status and prognosis was not available, our study suggests disparities in age at diagnosis in HNC patients based on rurality, race, and gender. Findings from this study could be used in identifying populations at risk of HNC, and could assist policy makers and health professionals in developing early cancer screening, education, and cancer care programs for the underserved high-risk populations in Alabama as well as in other parts of the United States.

## Health Equity Implications

Overall, majority of HNC patients present at the clinic at later stage (III/IV) of cancer, regardless of rurality. Understanding age, gender, race based on urban and rural residency is important when screening for HNC and targeting early intervention. In particular, young individuals may have different behavior patterns (e.g., smoking, drinking, and sexual practices) in urban and rural settings, specifically in the South, that may make them more susceptible to developing HNC.

## Abbreviations Used

CCC: Comprehensive Cancer Center
CI: confidence interval
CUP: carcinoma of unknown primary
EBR: Epstein Barr virus
HNC: head and neck cancer
HPV: human papillomavirus
ICD: International Classification of Diseases
IQR: interquartile range
NCI: National Cancer Institute
OPC: oropharyngeal cancer
OR: odds ratio
UAB: University of Alabama at Birmingham
